# N‑Doped Graphene for Biomedical Applications: A Comparative Biocompatibility Assessment of Green and Chemical Exfoliation

**DOI:** 10.1021/acs.chemrestox.5c00167

**Published:** 2025-09-05

**Authors:** Eirini Papanikolaou, Antrea- Maria Athinodorou, Michaela Patila, Panagiota Zygouri, Konstantinos Spyrou, Mohammed Subrati, Christina Alatzoglou, Evangelia Dounousi, Dimitrios P. Gournis, Konstantinos T. Kotoulas, Ming Xie, Andrew D. Burrows, Gareth Cave, Dimitrios Peschos, Konstantinos Tsamis, Lampros Lakkas, Haralambos Stamatis, Yannis V. Simos

**Affiliations:** † Department of Physiology, Faculty of Medicine, School of Health Sciences, 69156 University of Ioannina, Ioannina 45110, Greece; ‡ Nanomedicine and Nanobiotechnology Research Group, 37796 University of Ioannina, Ioannina 45110, Greece; § Nephrology Clinic, University Hospital of Ioannina, Ioannina 45110, Greece; ∥ Laboratory of Biotechnology, Department of Biological Applications and Technology, 37796 University of Ioannina, Ioannina 45110, Greece; ⊥ Department of Materials Science and Engineering, 69002 University of Ioannina, Ioannina 45110, Greece; #, School of Chemical and Environmental Engineering, Technical University of Crete (TUC), Chania, Crete GR-73100, Greece; ∇ Department of Chemical Engineering, 1555 University of Bath, Claverton Down, Bath BA2 7AY, United Kingdom; ○ Department of Chemistry, University of Bath, Claverton Down, Bath BA2 7AY, U.K.; ◆ School of Science and Technology, Nottingham Trent University, Nottingham NG11 8NS, U.K.

## Abstract

Graphene-based nanomaterials have transformed biomedical applications due to their exceptional physicochemical properties, and nitrogen (N)-doping further enhances the electrocatalytic activity of graphene. Driven by the demand for safer and more sustainable nanomaterials, in this work, we compared eco-friendly produced *N*- doped graphene (bD) with conventionally synthesized *N*- doped graphene (cD) in three different cell lines. Across all cell types and assays, cD was more toxic than bD. In NIH/3T3 fibroblast cells, cD activated the Nrf2 signaling pathway, whereas in HaCaT keratinocytes, it triggered oxidative stress responses and increased the apoptotic population. High doses of cD also affected THP-1-derived macrophages by inducing apoptosis and arresting the cell cycle in the G0/G1 phase. Although high doses of bD were also cytotoxic, overall, its effects were milder than cD. Our results confirm that green exfoliation of *N*- doped graphene retains its desirable biomedical properties while enhancing its biocompatibility, making bD a safer choice for future biomedical applications.

## Graphical abstract

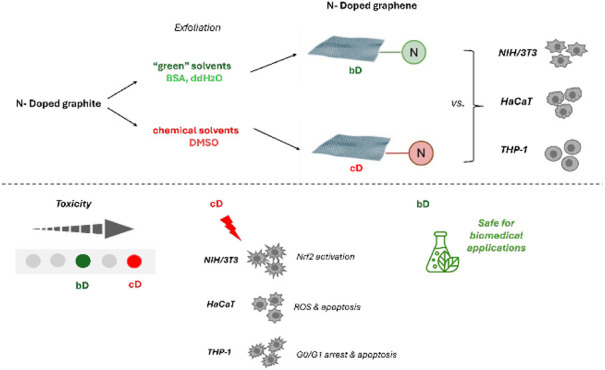

## Introduction

Nanomaterials have revolutionized the field of medicine by significantly improving existing clinical practices and addressing medical challenges that for years were impossible to address. They have also paved the way for new research opportunities and innovative biomedical applications.

Graphene has emerged as an extremely remarkable material among nanomaterials due to its unique physicochemical properties. It is a flexible, transparent, highly thermal conductive material^1^ with a significant tensile strength.^2,3^ It is regarded as the thinnest and strongest crystalline structure to date, and its carbon- carbon bonds are recognized as the strongest in nature.^4^ Graphene has transformed research across various fields, especially in medicine. Among other applications, graphene’s properties have greatly contributed to the development of high- precision biosensors for detecting biological agents^5^ and diagnostic devices have been developed based on these sensors.^6^ However, unlike other carbon nanomaterials, its exfoliation from graphite requires harsh, organic solvents, limiting its use in other biomedical applications, such as drug delivery in the human body, and raising concerns about its potential negative effects on the ecosystems. Unlike other graphene- based nanomaterials such as graphene oxide (GO), pristine graphene is hydrophobic^7^ and its hydrophobicity reduces its biocompatibility with human cells and tissues.^8^

The need to develop more biocompatible and environmentally friendly nanomaterials led researchers into synthesizing the so-called “green graphene”. This term includes environmentally friendly techniques that utilize biological molecules to exfoliate graphene from graphite, as well as ecological methods to reduce GO, using non- toxic reducing agents.^9,10^ Besides its eco- friendly composition “green” graphene is also characterized by its cost- effectiveness and low- energy requirements.^11^

Nanomaterials synthesized with ecological techniques have shown comparable efficacy to traditional chemical structures, across various biomedical applications. However, there is a notable lack of studies assessing the toxicity of these nanomaterials, leaving their clinical value uncertain. This challenge is common in the field of nanomedicine. Although many graphene-based nanomaterials have been developed and appear promising for biomedical applications, their biocompatibility and safety have not been sufficiently explored in preclinical studies. Consequently, the existing literature highlights a significant gap in understanding the potential risks and benefits of these materials.^12^

The toxicity of graphene nanomaterials is influenced by many physicochemical factors, including their size, shape, edge sharpness, nanopores, π–π bonds, surface charge, conductivity, hydrophilicity/hydrophobicity and surface functional modification.^13^ As various synthesis strategies have emerged and different molecules are employed to modify the surfaces of graphene nanomaterials functionally, their toxicity can vary greatly. Moreover, the biological response of these nanomaterials to living cells and organisms seems to depend on the specific types of cells and tissues they encounter.^14^ Therefore, it is crucial to conduct comprehensive *in vitro* and/or *in vivo* investigations of their biocompatibility, before applying any graphene- based nanostructure in biomedical applications.^15^

We have previously examined the toxicity of pristine graphene across three different cell lines, as well as the biocompatibility of green-synthesized graphene (“biographene”), derived from the exfoliation of graphite using biological solvents as stabilizing and modifying agents.^12^ We found that biographene was safer at a high dose range than its chemical counterpart. In this study, we assessed the biocompatibility of *N*- doped graphene, derived from exfoliation of N-doped graphite via organic solvent (cD) and green-synthesized N-doped graphene, derived from exfoliation of N-doped graphite using biological solvents in water (bD). Since N-doping enhances the electrocatalytic property of graphene,^16^ producing this nanomaterial through safer methods could pave the way for new biomedical applications.

## Materials and Methods

### Chemicals and Reagents

Graphite, Dulbecco’s Modified Eagle’s Medium High glucose, RPMI-1640 Medium, Phosphate Buffer Saline (PBS), Thiazolyl blue tetrazolium bromide (MTT), 2′-7′-dichlorofluorescein diacetate, ≥ 97% (DCFDA), Crystal Violet, Glycine, Trizma base, Sodium dodecyl sulfate, Phenylmethylsulfonyl fluoride (PMSF), Ammonium persulfate, Protease and phosphatase inhibitor cocktail, Laemmli- Lysis buffer and Bovine serum albumin (BSA, 98% Fraction V) were purchased from Sigma-Aldrich Chemical Co. (St. Louis, MO, USA). Fetal bovine serum (FBS) was obtained from PAN BIOTECH (Aidenbach, Germany). Trypsin-EDTA, Penicillin- Streptomycin and L- Glutamine were purchased from Biowest (Riverside, CA, USA). Hanks’ Balanced Salt Solution (HBSS) was obtained from Biosera (Nuaille, France). FITC Annexin V, Annexin V Binding buffer and Propidium Iodide (PI) were purchased from BioLegend Inc. (San Diego, CA, USA). Triton X, Tween 20 and Glycerol were purchased from Thermo Fisher Scientific Inc. (Waltham, MA, USA). Precision Plus Protein Dual Color Marker and Clarity Western ECL Substrate were purchased from Bio-Rad Laboratories, Inc. (Hercules, CA, USA). Primary rabbit monoclonal antibodies to HO-1 (1:1000) to NF-kB (1:1000) and to NRF2 (1:750), as well as secondary rabbit-specific horseradish peroxidase-conjugated antibody (1:1000), were all purchased from Cell Signaling Technology, Inc. (Danvers, MA, USA). Primary mouse monoclonal antibody to a- tubulin (1:1000) and secondary mouse-specific horseradish peroxidase-conjugated antibodies (1:1000) were obtained from Santa Cruz Biotechnology (Santa Cruz, CA, USA). All the aqueous solutions were prepared by double-distilled water (ddH_2_O).

Expanded graphite was derived upon rapid microwave heating for about 5 min at 600 W.

### Synthesis of *N*- Doped Expanded Graphite (EG)

In a typical synthesis, the N-doped graphite sample was prepared by slowly adding 50 mL of an ethanolic solution of urea (324 mM) to 1 g of expanded graphite (EG) while stirring. The resulting dispersion was stirred for 2 h at room temperature (RT) and then evaporated at 60 °C to obtain a thick paste. After that, the paste was dried in the oven overnight, and then pyrolyzed at 900 °C for 4 h with a heating rate of 10 °C min^–1^ and under nitrogen flow. Finally, the obtained mass was washed several times with ethanol and then dried overnight at RT.^17^

### Liquid Exfoliation of cD and bD

Liquid exfoliation of cD to produce bD was based on our previous work.^18^ Briefly, 100 mg of cD was dispersed to 20 mL of ddH_2_O by ultrasonication for 1 h (200 W, 10 kHz, pulse 50%). Next, 5 mL of an aqueous solution containing 100 mg BSA was added to the cD dispersion and the mixture was stirred for 1 h at 25 °C. The unexfoliated nanomaterial was separated by centrifugation (2500 rpm, 10 min), and the supernatant was collected carefully to acquire the lightest flakes. The supernatant was then subjected to three more centrifugations (16000 rpm, 60 min) to remove the excess BSA. At the end of each centrifugation, the supernatant was disposed of, and the pellet, containing the resulting bD, was dispersed in ddH_2_O. Finally, 1 mL aliquot of the redispersed pellet was freeze-dried.

Chemically exfoliated N-doped graphene was derived by sonicating N-doped expanded graphite in DMSO solution for 6 h. The final solution was centrifugated in 1000 rps and the supernatant was collected.

### Cell Lines

When it comes to biomedical applications, toxicity of nanomaterials should be assessed primarily in cell types that are abundant in the human body and more likely to interact with foreign materials, such as fibroblasts, skin-specific cells, and immune cells.^19^ For this reason, this study utilized three different cell lines; a fibroblast cell line isolated from a mouse NIH/Swiss embryo (NIH/3T3 cell line, ATCC, CRL-1658), an immortal keratinocyte cell line derived from adult human skin (HaCaT cell line, CLS GmbH, 300493), and a human monocyte cell line obtained from a patient with acute monocytic leukemia (THP-1 cell line, DSMZ, ACC16). NIH/3T3 and HaCaT cells were cultivated in high glucose Dulbecco’s modified eagle medium supplemented with 10% (v/v) fetal bovine serum (FBS), 1% (v/v) l-glutamine and 1% (v/v) penicillin- streptomycin solution. THP-1 monocytes were cultivated in RPMI- 1640 medium supplemented with 10% (v/v) fetal bovine serum (FBS), 1% (v/v) l-glutamine and 1% (v/v) penicillin- streptomycin solution. All cell lines were maintained in a humidified incubator (5% CO_2_, 95% air), at 37 °C. Prior to all experiments, THP-1 monocytes were differentiated into mature macrophages using phorbol 12-myristate 13-acetate (PMA) at a concentration of 100 ng/mL for 24 h.^20^

### Cell Viability Assay

A total of 5 × 10^3^ cells/well of HaCaT and NIH/3T3 cells along with 4 × 10^4^ cells/well of differentiated THP-1 macrophages, were seeded in a 96- well microplate and incubated for 24h at 37 °C with 5% CO_2_. Following this, cells were treated with increasing concentrations (0.5–200 μg/mL) of either bD or cD for 24 and 48 h. Cell viability was assessed after adding 3-(4,5-dimethylthiazol-2-yl)-2,5-diphenyltetrazolium bromide solution (MTT) for 3 h. The formazan that was formed, was dissolved in dimethyl sulfoxide (DMSO) and the optical density of the living cells was measured at 570 nm, with a background reading at 690 nm, using a microplate spectrophotometer (Infinite 200 Pro, Tecan, Switzerland). All experiments were conducted in triplicate for each condition and percentages of cell viability above 80% were considered as non- toxic.^21^

### Clonogenic Assay

As clonogenic assay can be performed only against adherent cell lines that proliferate,^22^ THP-1-derived macrophages were excluded from this study. Thus, NIH/3T3 and HaCaT cells were seeded in 6- well plates at a density of 1 × 10^3^ cells/well and incubated for 24 h in a humidified incubator (5% CO_2_, 95% air, 37 °C). Cells were then treated with selected concentrations (1, 10, 20, 50, and 100 μg/mL) of bD and cD for 48 h. A week later, cells were washed once with PBS and stained with a dye mixture consisting of 0.5% w/v crystal violet, 6% v/v glutaraldehyde and ddH_2_O. The number of visible colonies was counted using the OpenCFU open-source software (version 3.9.0)^23^ and the surviving fraction (SF) of the treated cells was calculated.^24^ All experiments were conducted in triplicate.

### Reactive Oxygen Species (ROS) Assay

To detect the presence of total free- ROS, the DCFH- DA staining was used. First, 15 × 10^4^ cells/well of HaCaT and NIH/3T3 cells, and 3 × 10^5^ cells/well of THP-1 derived macrophages were seeded in 6- well plates. Once the cells attached to the plates, selected doses (20, 50, and 100 μg/mL) of either bD or cD were added to the medium, for 24 h. After treatment’s incubation period, cells were detached with trypsin, washed once with PBS and centrifuged at 3000 rpm for 5 min. Cell pellets were resuspended in 2 mL cold Hanks’s Balanced Salt Solution (HBSS) containing 2.5 μM of 2′,7′-Dichlorodihydrofluorescein diacetate (DCFH-DA) and incubated for 30 min, at 37 °C in the dark. After incubation, the samples were stained with Propidium Iodide (PI) and placed on ice. ROS production of the treated and untreated cells was measured directly by flow cytometry (Partec ML, Partec GmbH, Leipzig, Germany). All experiments were conducted in triplicates.

### Detection of Apoptosis

Five × 10^4^ cells/well of NIH/3T3 and HaCaT cells and 24 × 10^4^ cells/well of THP-1 derived macrophages were seeded in 48- well plates and incubated in a humidified incubator (5% CO_2_, 95% air, 37 °C) for 24 h. The medium of the cells was then renewed with fresh medium containing increasing doses (20, 50, and 100 μg/mL) of either bD or cD for 24 h. On the day of the processing, cells were dissociated from the plates with trypsin and the number of cells on each well was calculated with a Neubauer hemocytometer. One × 10^5^ cells of each well were then transferred into clean Eppendorf tubes and were centrifuged. Cell pellets were resuspended in 100 μL Annexin V Binding buffer, stained with FITC Annexin V and PI and incubated at RT for 15 min, in the dark. After incubation, 400 μL of Annexin V binding buffer was added to the samples and they were analyzed immediately on a flow cytometer (Partec ML, Partec GmbH, Leipzig, Germany). All experiments were conducted in triplicates.

### Cell Cycle Analysis

One × 10^5^ cells/well of NIH/3T3 and HaCaT cells and 5 × 10^5^ of PMA- treated THP-1 cells were seeded in 6- well plates and incubated for 24 h in a humidified incubator (37 °C, 5% CO_2_, 95% air). The following day, the culture medium was renewed with fresh medium containing increasing doses (20, 50, and 100 μg/mL) of either bD or cD. After 24 h, the cells were trypsinized, centrifuged, and the resulting pellets were washed once with ice- cold PBS. Pellets were then resuspended in 0.5 mL ice- cold PBS, and 0.5 mL of absolute ethanol was added to the solution dropwise. The samples were kept frozen at −20 °C for 7 days. The day of the processing, samples were centrifuged to eliminate the absolute ethanol, and pellets were then resuspended in 1 mL fresh ice-cold PBS. PI (25 μg/mL) and RNaseA (25 μg/mL) were added, and the samples were incubated at 37 °C for 30 min in the dark. The samples were placed on ice and immediately analyzed by flow cytometry (Partec ML, Partec GmbH, Leipzig, Germany). All experiments were conducted in triplicates.

### Western Blotting Analysis

NIH/3T3, HaCaT and THP-1 cells were grown in 10 cm culture dishes and were treated with three doses (20, 50, and 100 μg/mL) of either bD or cD, for 24 h. After treatment’s incubation period, cells were washed twice with ice- cold PBS and placed on ice. Then, 7 mL of ice- cold PBS was added to the plates, and the adherent cells were harvested mechanically using a cold plastic cell scraper. After centrifugation at 11000 rpm for 8 s, cell pellets were resuspended in 1 mL ice- cold lysis buffer (RIPA buffer) supplemented with protease and phosphatase inhibitors, and left on ice for 20 min. During this incubation and every 5 min, the samples were resuspended with a 21 G × 1 1/2 needle-syringe and vortex. The samples were then sonicated on ice for 20 s and were centrifuged at 14800 rpm for 20 min, at 4 °C. Supernatants (i.e., the total cell protein lysate) were collected into clean Eppendorf tubes, and the protein concentration in each sample was measured using the Pierce BCA Protein Assay Kit (Thermo Fisher Scientific Inc., Rockford, IL, USA). Equal amounts of protein from each sample were loaded onto a 10% sodium dodecyl sulfate-polyacrylamide (SDS- PAGE) gel and electrophorized. Following electrophoresis, the proteins were transferred to nitrocellulose membranes. The membranes were blocked with 5% nonfat milk in Tris-buffered saline (TBS) containing 1% Tween 20 (TBST) for 1 h, at RT. The membranes were then incubated overnight at 4 °C, with the desired primary antibody diluted in 5% nonfat milk in TBST. After three quick washes with TBST (3 × 5 min), the membranes were incubated with the proper secondary antibody diluted in the same blocking solution, for 1 h at RT. Following three additional washes with TBST (3 × 5 min), the membranes were treated with an enhanced chemiluminescence (ECL) substrate (Clarity Western ECL Substrate, #1705061, Bio-Rad Laboratories, CA, USA), for 5 min. The blots were captured using the ChemiDoc MP Imaging System (Bio-Rad Laboratories, CA, USA) and analyzed with ImageLab software (Bio-Rad Laboratories, CA, USA). All experiments were conducted in triplicates.

### X-ray Photoelectron Spectroscopy (XPS)

XPS measurements were conducted under ultrahigh vacuum (UHV) conditions with a base pressure of 5 × 10^– 1 0^ mbar, using a SPECS GmbH system equipped with a monochromatic Mg Kα X-ray source (hν = 1253.6 eV) and a Phoibos 100 hemispherical analyzer. Samples were mounted on suitable substrates and pre-equilibrated in high vacuum before being introduced into the main analysis chamber. The energy resolution was set to 1.18 eV, and spectra were acquired at a photoelectron takeoff angle of 45° relative to the surface normal. Each spectrum represented the average of five scans, with an energy step size of 0.05 eV and a dwell time of 1 s per point. Binding energies were referenced to the C 1s core level at 284.6 eV. Spectral processing included Shirley background subtraction and peak deconvolution using mixed Gaussian–Lorentzian functions, implemented in a least-squares fitting routine (WinSpec).

### Transmission Electron Microscopy (TEM)

Transmission electron microscopy (TEM) was performed using a JEOL 2100 Transmission Electron Microscope (JEOL, Tokyo, Japan), equipped with a Gatan Rio16 4k*4k CMOS camera. Carbon-coated copper grids (Agar Scientific, UK) were plasma-coated (Q300T D Plus, UK) prior to dropcasting. The suspension of graphitic fragments in water (10 μL) were loaded onto the grids, which were air-dried at room temperature for 15 min. Excess suspension was bloated away prior to imaging. Postimaging, interlayer *d*-spacing was quantified using the ImageJ (Fiji) software.

### Zeta Potential

Zeta potentials of the graphitic fragments were measured using a Malvern Zetasizer Ultra (ZSU3305), using a folded capillary cell (DTS1070). Measurements were conducted at 25 °C.

### Statistical Analysis

All data were expressed as mean values ± standard deviation. The statistical significance difference between two mean values was assessed using the two- sample *t*- test. A *p*- value of less than 0.05 (*p* < 0.05) was considered statistically significant.

## Results

### X-ray Photoelectron Spectroscopy

X-ray photoelectron spectroscopy was applied to attest the successful doping of nitrogen in the EG structure. Survey in Figure 1a reveals the characteristic peaks of N-doped graphite such as Carbon, Oxygen and Nitrogen. The presence of nitrogen is proof of successful doping in the main graphitic carbon frame. From the high-resolution C 1s photoelectron peak in Figure 1b, we detect 5 peaks. The main peak at 284.5 eV is due to the main C = C framework of the graphitic structure, while the peak at 290 eV is derived from the N–C = O bond.^25^ The lack of pi-pi* interaction is evidence of successful doping. The presence of nitrogen can disrupt the continuous π-conjugation of the graphene sheet, potentially affecting the pi-pi* transitions.^26^ Moreover, the peak at 286.0 eV is due to the C–N bonds.^27–29^ There are two more peaks owing to oxygen functionalities and more specifically C–O–C and C = O bonds, which were created during the synthetic procedure for the nitrogen doping of graphite.

**Figure 1:**
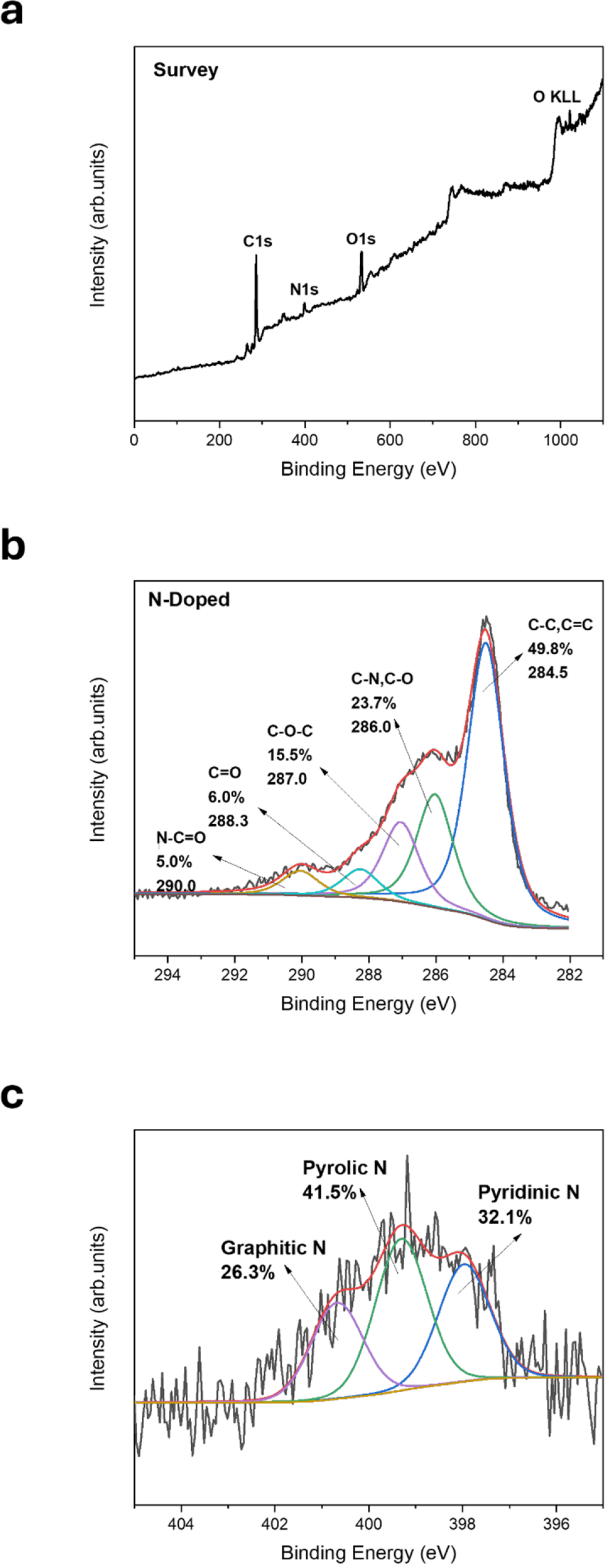
Survey XPS (a), Carbon 1s photoelectron spectra (b) and Nitrogen 1s photoelectron spectra (c) of N-doped graphite.

The N 1s photoelectron peak gives information about the type of doping on the expanded graphite.^25,30^ From the deconvolution of the N 1s peak, we receive three types of doping, pyridinic, pyrolic, and graphitic nitrogen, as depicted in Figure 1c.

### Structural and Colloidal Properties of N-Doped Graphene

Transmission electron microscopy (TEM) revealed distinct structural differences between the N-doped graphene samples, which appear strongly influenced by the exfoliation technique employed (Figure 2). The larger, chemically exfoliated fragments (cD, > 30 nm length) predominantly presented an edge-on orientation, showing well-ordered stacked layers. For these samples, the measured interlayer spacing, corresponding to the (002) plane, was 0.318 ± 0.006 nm, which is within the experimental error of pristine graphite (0.335 nm), suggesting a highly ordered structure. The smaller, biologically exfoliated fragments (bD, < 6 nm length) typically lay flat on the TEM grid, presenting a top-down view of the graphitic lattice. These samples exhibited a *d*-spacing of 0.25–0.28 nm, which corresponds to the in-plane (100) lattice spacing.

**Figure 2:**
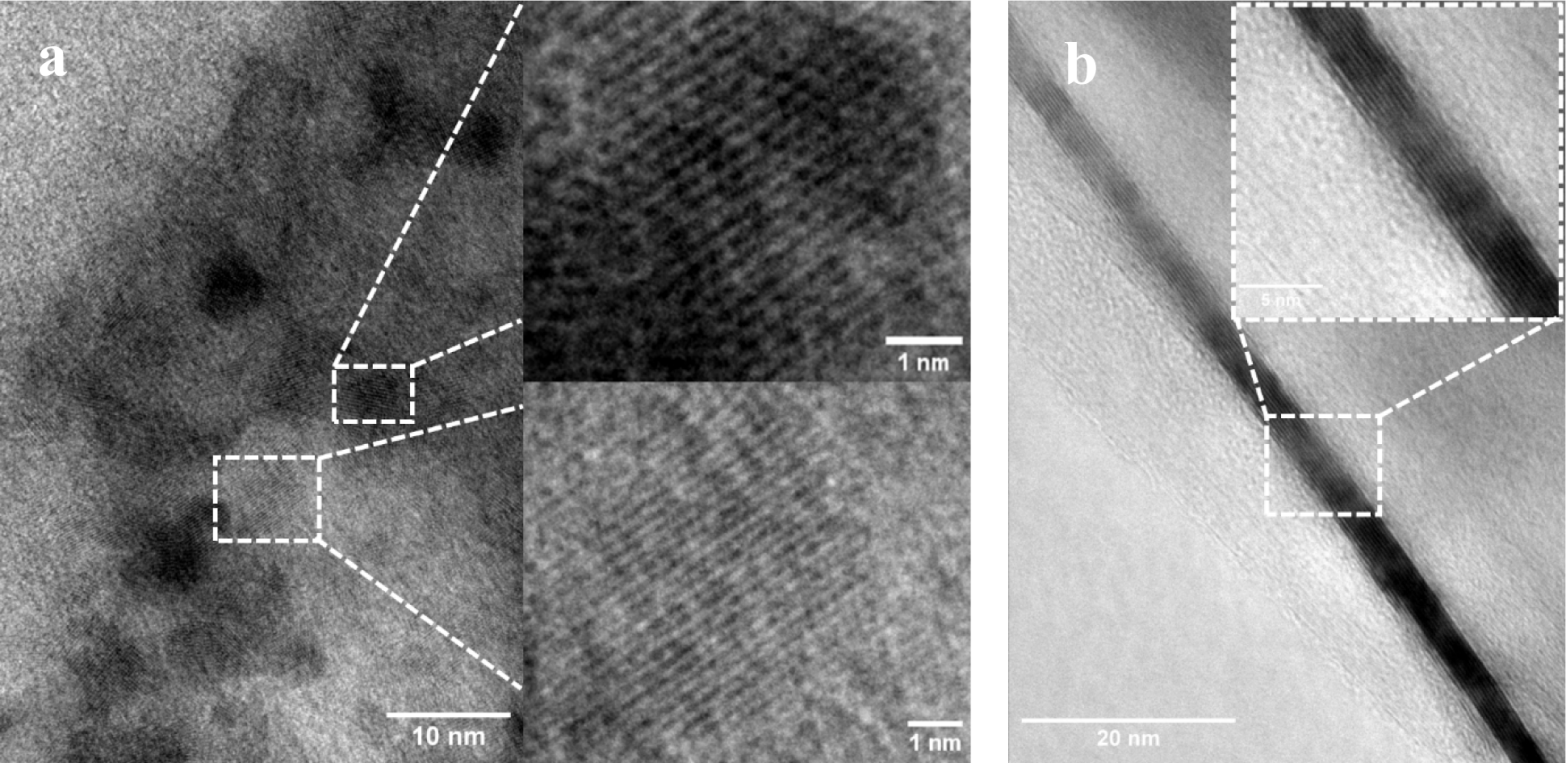
Transmission electron microscopy images of N-doped graphene samples exfoliated via biological (bD) and chemical (cD) methods. (a) bD sample showing small, sheet-like graphitic fragments with reduced interlayer *d*-spacing. (b) cD sample displaying larger, multilayered tube-like structures with expanded interlayer distances. Insets highlight lattice fringes, demonstrating the morphological and structural differences arising from the two exfoliation techniques. Interlayer spacing was measured using ImageJ (Fiji) software.

This analysis shows that the two exfoliation methods produce not only different fragment sizes but also different predominant morphologies, leading to the observation of distinct crystallographic planes. The chemical exfoliation appears to yield larger, ribbon-like structures that settle on their sides, while the biologically mediated process produces quasi-zero-dimensional fragments that lie on their basal plane. The compact interlayer spacing of cD suggests the exfoliation is efficient without introducing significant disorder or functional groups that would typically expand the layers, as is common in graphene oxide (GO) systems.^31^ Conversely, the clear in-plane lattice spacing of bD confirms the high crystallinity of these ultrasmall fragments.^32^

Notably, TEM also revealed distinct morphological differences based on the exfoliation method. The cD samples frequently formed tube-like structures, likely resulting from rolling and folding of graphene layers during chemical exfoliation in DMSO, a solvent known for effective intercalation and exfoliation.^33^ In contrast, bD samples exhibited more irregular, sheet-like morphologies with diverse lateral sizes, consistent with gentler, protein-assisted exfoliation using BSA.

These structural differences translated into varied colloidal behaviors, as confirmed by zeta potential measurements (Figure 3). The cD sample displayed a single negative peak at – 7.2 mV, suggesting moderate electrostatic stability. Meanwhile, bD exhibited a broader zeta distribution with a main peak at – 1.5 mV and a secondary peak around – 19.1 mV. This suggests heterogeneity in the bD surface charge, likely reflecting complex interactions between the graphene surface and BSA during green exfoliation, via π–π stacking, electrostatic interactions and hydrogen bonding.^34^ This surface heterogeneity may arise from nonuniform adsorption of BSA to the graphitic system.^35^ While lower zeta values (closer to 0 mV) typically indicate decreased colloidal stability, the presence of the strongly negative subpopulation in bD may still contribute to its suspension stability. Importantly, the difference in surface charge between cD and bD reflects differences in surface chemistry, which are known to influence nanomaterial–cell interactions, including cellular uptake, membrane binding, and intracellular responses.^19^

**Figure 3:**
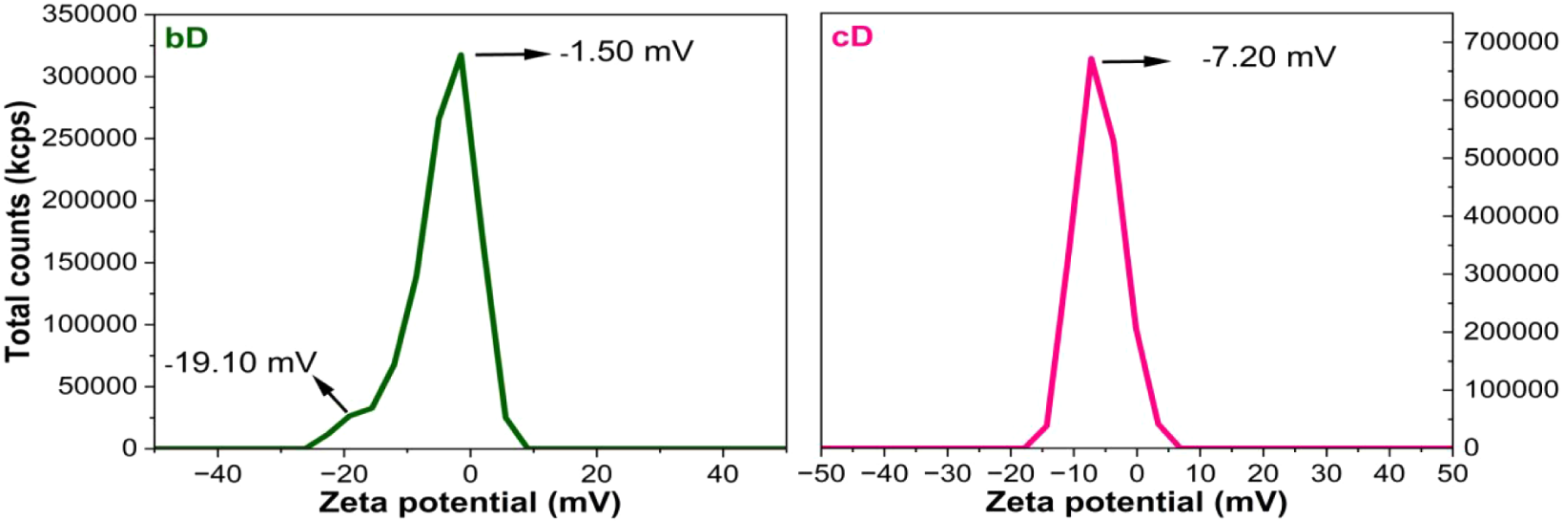
Zeta potential distribution of biologically (bD) and chemically (cD) exfoliated N-doped graphene. The bD sample exhibits a broad surface charge profile with a dominant peak at – 1.5 mV and a secondary peak at – 19.1 mV, suggesting heterogeneous surface functionalization due to protein–graphene interactions during green exfoliation. In contrast, the cD sample shows a single, more uniform peak at – 7.2 mV, indicating a relatively homogeneous surface chemistry resulting from chemical exfoliation in DMSO.

### ΜΤΤ Assay

Exposure of NIH/3T3 cells for 24 h to the lowest dose of cD (0.5 μg/mL) resulted in a 30% reduction in cell viability, indicating cytotoxicity. At 200 μg/mL viability dropped to 50%. On the contrary, bD was nontoxic up to 10 μg/mL, as cell viability remained high, at 95%. At higher doses (50–200 μg/mL), bD induced a mild dose-dependent toxicity, and viability dropped to 50–55% (Figure 4a). cD did not exhibit time-dependent toxicity on NIH/3T3 cells as cell viability at 48 h was comparable to that of 24 h. bD showed also non- significant time- dependent toxicity (Figure 4b).

**Figure 4:**
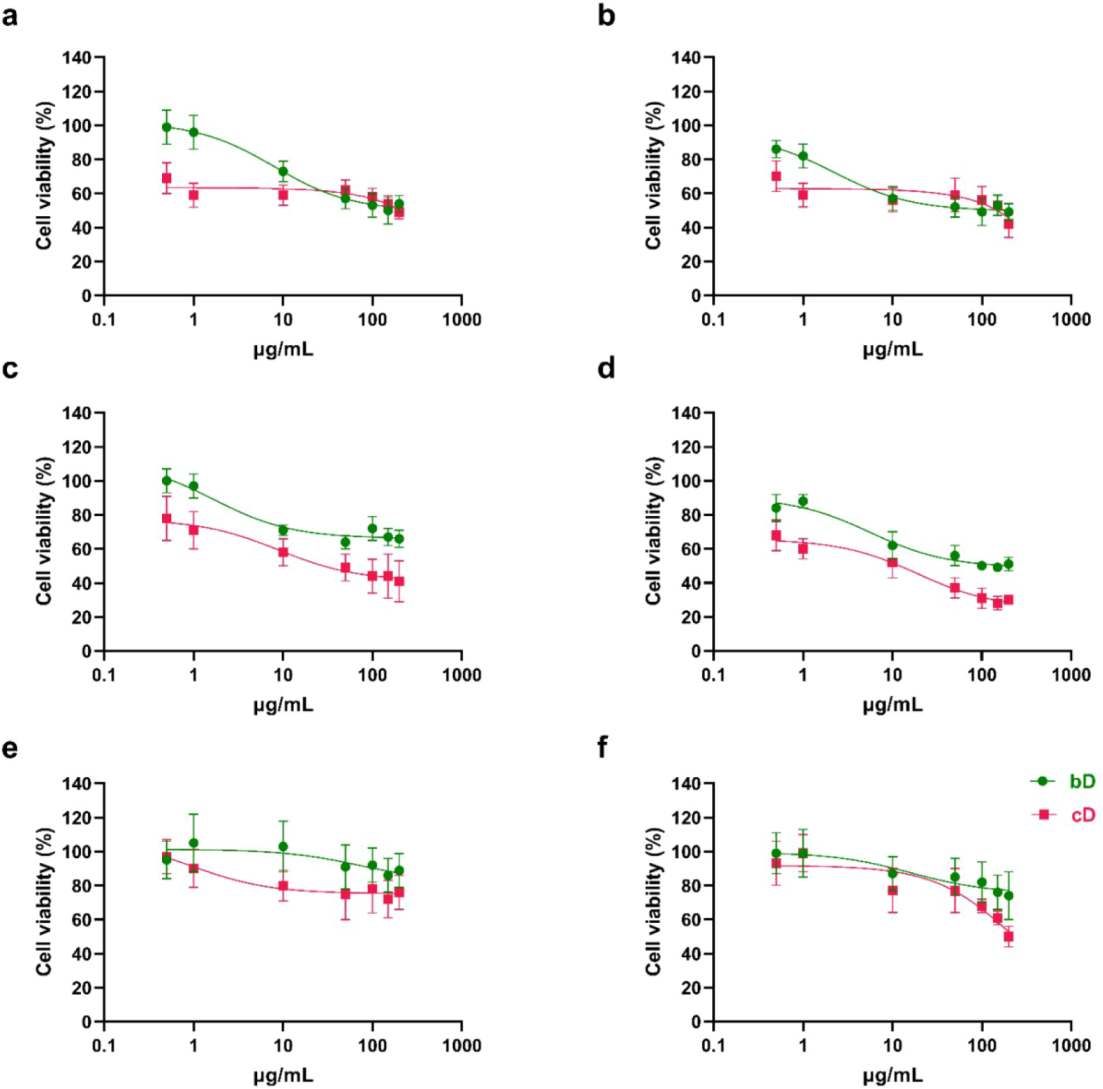
Cell viability of NIH/3T3 (a,b), HaCaT (c,d) and THP-1 derived macrophages (e,f) after treatment with increasing doses of bD and cD for 24 (a,c,e) and 48 h (b,d,f).

Both nanomaterials induced a dose- dependent toxicity in HaCaT cells, but at all tested doses, cD was more toxic than bD, at both 24 and 48 h. bD was non- toxic to cells up to the dose of 1 μg/mL, as cell viability remained up to 85%. At 10 μg/mL, viability of cells reduced to 70%, for both 24 and 48 h. Above this dose, a drop at 65% and 50%, for 24 (Figure 4c) and 48 h (Figure 4d), respectively, was observed. For cD, the viability of cells was below 75% at the lowest tested dose of 0.5 μg/mL. At higher doses, cell viability reduced significantly and reached 40% at 24 h (Figure 4c) and 30% at 48h (Figure 4d).

Treatment with bD for 24 h showed no toxic effects in THP-1 derived macrophages, with cell viability exceeding 90% (Figure 4e). At 48 h bD was non- toxic up to 100 μg/mL. Cell viability dropped to 75% at the two highest doses of 150 and 200 μg/mL (Figure 4f). At 24 h, cD was non- toxic up to 10 μg/mL, and then exhibited a mild toxicity, as cell viability reduced to 75% (doses from 50 to 200 μg/mL) (Figure 4e). However, treatment with cD for 48 h, resulted in a significant dose- dependent toxicity, where cells’ viability dropped remarkably and reached 50% at the maximum dose (Figure 4f).

### Colony Forming Efficiency Assay

Low doses of bD did not significantly affect NIH/3T3 cells’ ability to form colonies. Surviving fraction (SF) was 0.9 at the lowest tested dose of 1 μg/mL and then reduced to 0.75 at 50 μg/mL. At the highest dose of 100 μg/mL, a significant decline on the ability of cells to form colonies was observed, and SF dropped to 0.65. Low doses of cD also did not affect NIH/3T3 cells’ colonies. Up to the dose of 50 μg/mL, the SF of cells exposed to cD was similar to that of cells exposed to bD. At 100 μg/mL, a significant reduction of colonies was observed. However, at this dose, cD ‘s effect was more intense than bD’s, as SF dropped to 0.45 (Figure 5a).

**Figure 5:**
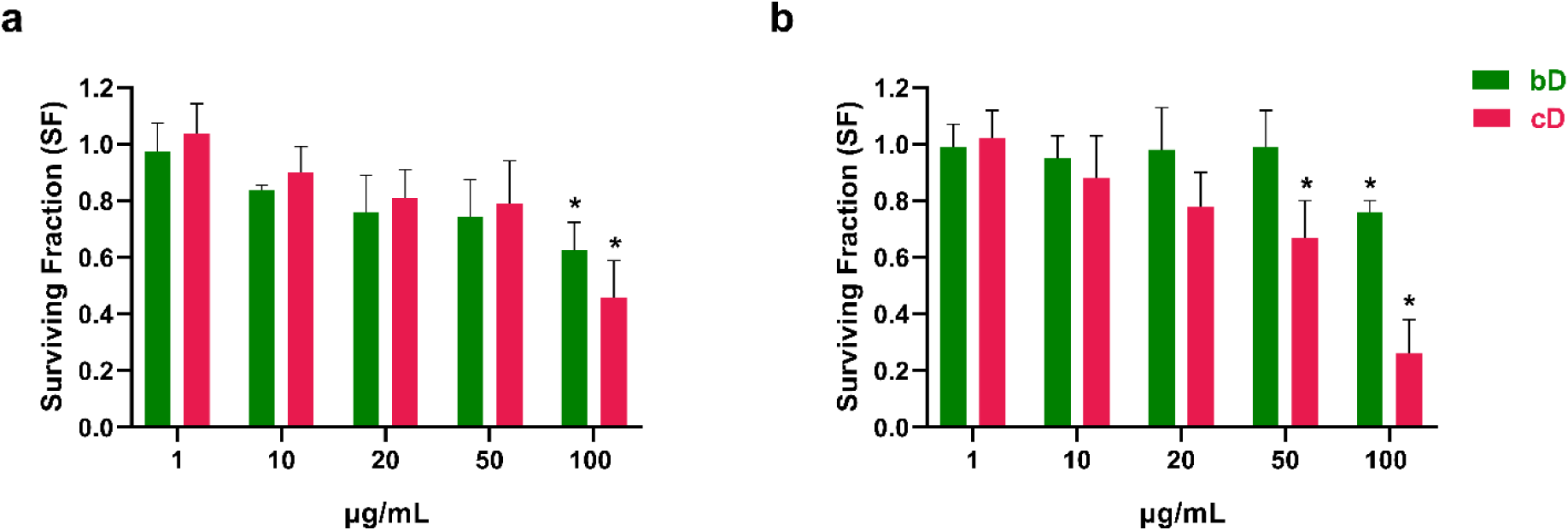
Clonogenic assay in NIH/3T3 (a) and HaCaT (b) cells after treatment with increasing doses of bD and cD for 48 h. *: Statistically significant difference compared to control (*p* < 0.05).

In HaCaT keratinocytes, bD exhibited effects similar to those observed in NIH/3T3 cells. Up to 50 μg/mL it did not affect HaCaT cells’ ability to form colonies. At 100 μg/mL of bD a statistically significant reduction in colonies was observed, as SF dropped to 0.75. On the contrary, cD affected HaCaT’s colonies in a dose-dependent manner. SF gradually declined to 0.65 at 50 μg/mL and reached 0.26 at the highest (Figure 5b)

### Intracellular ROS Production

Treatment with either bD or cD had the same effect in ΝΙΗ/3Τ3 cells, inducing a 10% increase in ROS production. This elevation in ROS was neither dose- dependent nor statistically significant (Figure 6a).

**Figure 6:**
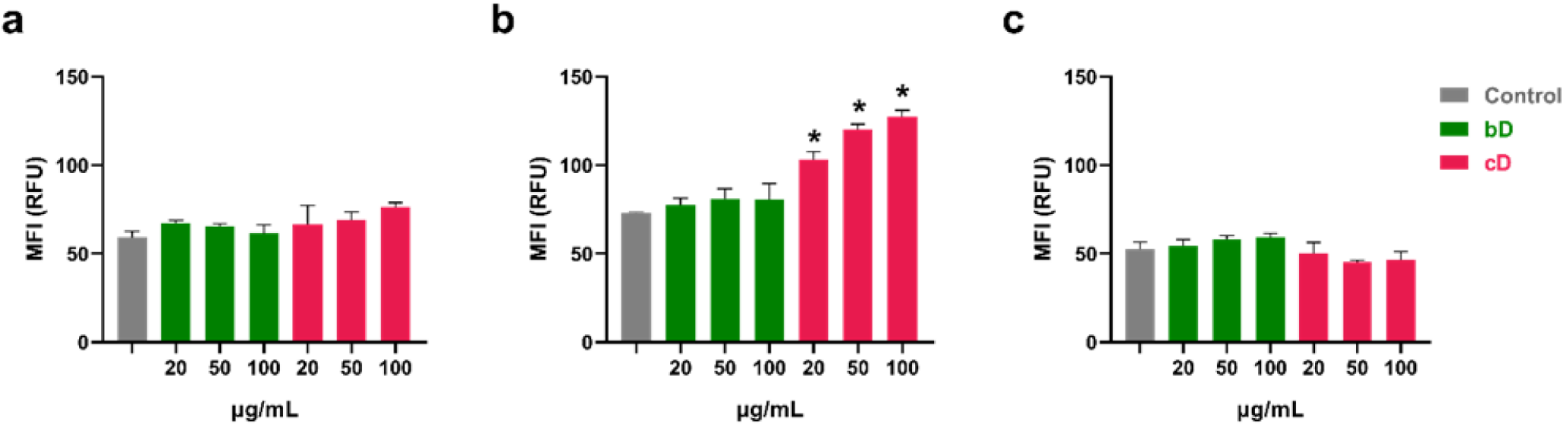
ROS formation in NIH/3T3 (a), HaCaT (b) and THP-1 derived macrophages (c) after treatment with increasing doses of bD or cD for 24 h.

bD had the same effect also in HaCaT cells. At all tested doses, an elevation of 10% in ROS formation was observed. However, cD induced a significant, dose-dependent elevation of ROS production in human keratinocytes. At the lowest dose of 20 μg/mL, cD induced a 41% increase in ROS formation, whereas at the highest doses of 50 and 100 μg/mL elevation of ROS was by 64% and 74%, respectively (Figure 6b).

Both nanomaterials had no impact on ROS levels in THP-1 derived macrophages (Figure 6c).

### Evaluation of Apoptosis

bD treatment for 24 h had no effect on NIH/3T3 cells’ population (Figure 7a-c). cD caused a slight increase in apoptosis of 4% at 20–50 μg/mL (Figure 7a,b) and 8% at 100 μg/mL (Figure 6c).

**Figure 7:**
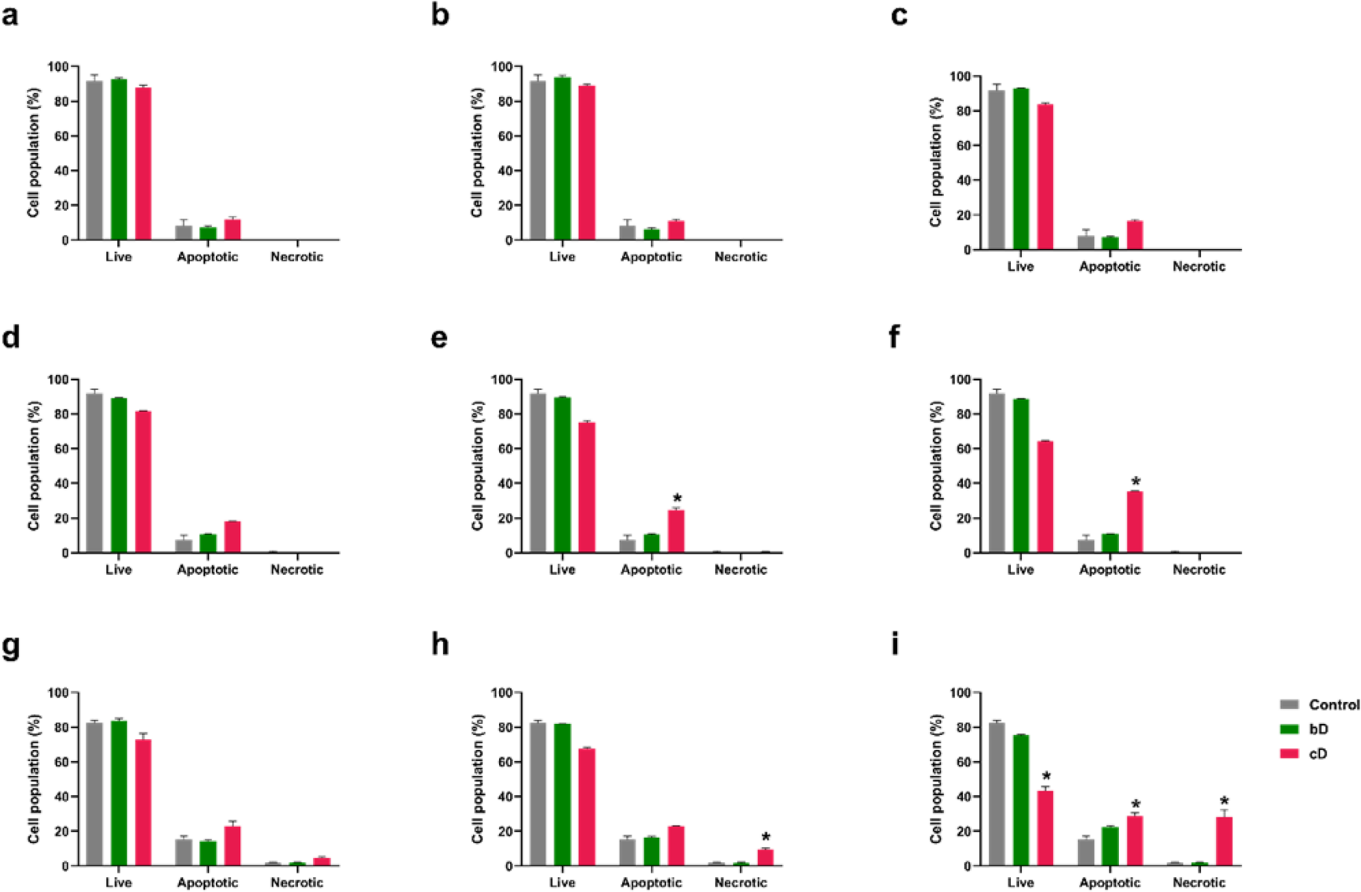
Live, apoptotic and necrotic cells (% cell population) in NIH/3T3 (a,b,c), HaCaT (d,e,f) and THP-1 derived macrophages (g,h,i), after treatment with 20 μg/mL (a,d,g), 50 μg/mL (b,e,h) and 100 μg/mL (c,f,i) of bD or cD for 24 h. *: Statistically significant difference compared to control cells (*p* < 0.05).

All tested doses of bD also did not affect the population of HaCaT cells. On the contrary, cD significantly impacted on the cell population. Treatment with 20 and 50 μg/mL of cD, increased the apoptotic cell population by 10% and 17%, respectively (Figure 7a,b). At the highest tested dose of 100 μg/mL, 28% of the cell population underwent apoptosis (Figure 7c).

In THP-1 derived macrophages, the highest dose of bD induced a small, nonstatistically significant increase by 7% in the apoptotic cell population (Figure 7i). However, cD affected the population of THP-1 cells, in a dose-dependent way. Treatment with 20 and 50 μg/mL of cD resulted in an increase of the apoptotic cell population of 8% (Figure 7g-h). Also, at the dose of 50 μg/mL necrotic population increased by 9% compared to control (Figure 7h). At the highest dose of 100 μg/mL the apoptotic cells increased by 13% and the necrotic cells by 28% compared to control (Figure 7i).

### Cell Cycle Arrest

At 20 and 50 μg/mL bD and cD had no significant effect on NIH/3T3 cells’ cell cycle (Figure 8a,b). However, at 100 μg/mL both graphene forms induced cell cycle arrest in S phase, increasing the S- phase population from 27 ± 6% (control), to 38 ± 4% with bD and to 39 ± 3% with cD (Figure 8c).

**Figure 8:**
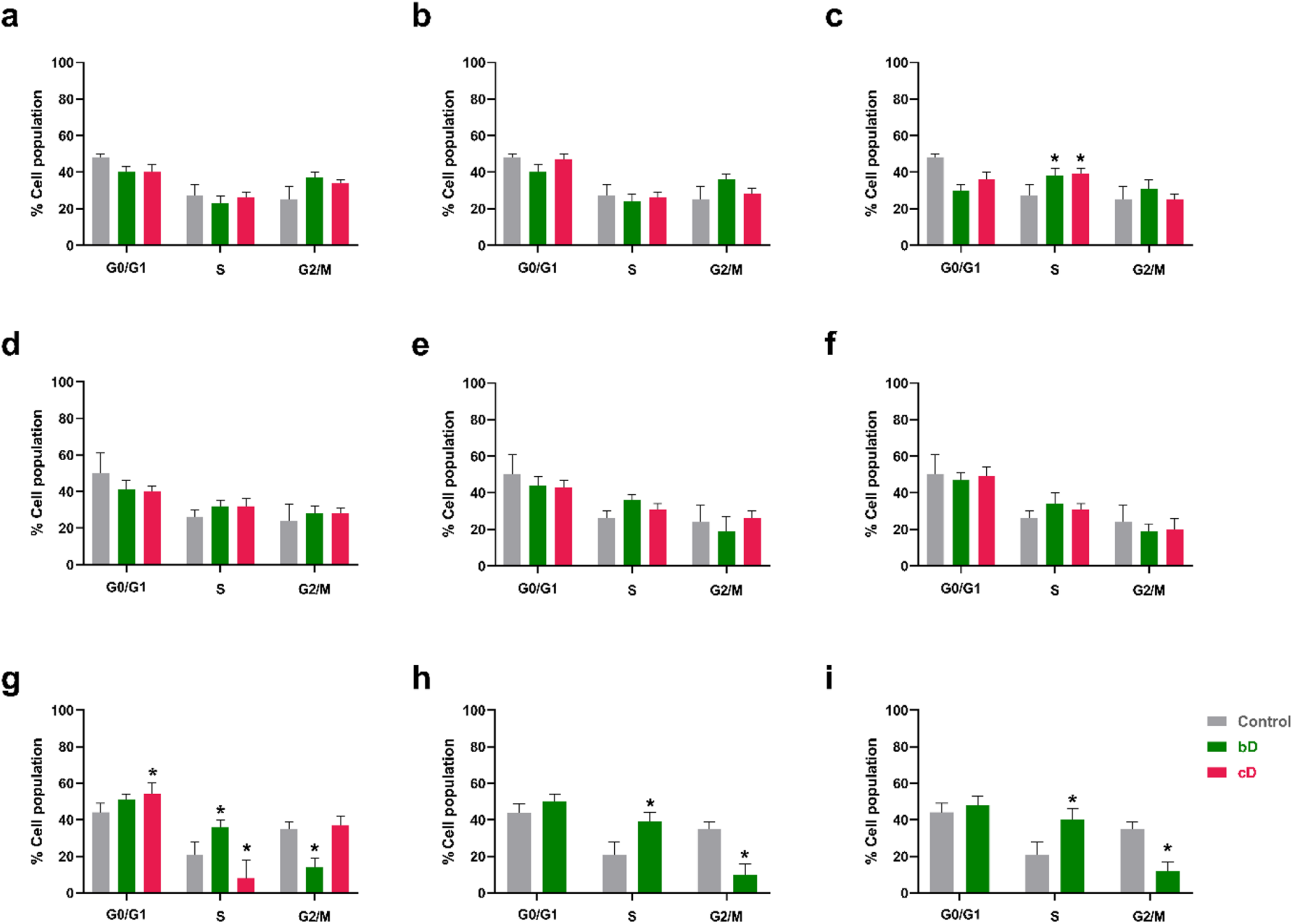
Cell cycle arrest in NIH/3T3 (a-c), HaCaT (d-f) and THP- derived macrophages (g-i) after treatment with 20 (a,d,g), 50 (b,e,h) and 100 μg/mL (c,f,i) of bD or cD for 24 h.

Both nanomaterials induced a slight but nonstatistically significant increase in S- phase also in HaCaT cells. At 100 μg/mL, the population of cells in G0/G1 phase reduced from 50 ± 13% (control), to 47 ± 4% after treatment with bD and to 49 ± 5% after treatment with cD. Accordingly, the population in S phase increased from 26 ± 4% in control cells, to 34 ± 6% in cells treated with bD, and to 31 ± 3% in cells treated with cD (Figure 8f).

Unlike the other two cell lines, bD and cD had a notable impact on the cell cycle of THP-1-derived macrophages. At 20 μg/mL they exhibited opposing effects. bD caused cell cycle arrest in S phase, increasing the S- phase population from 21 ± 7% (control) to 36 ± 4%. On the contrary, cD induced cell cycle arrest in the G0/G1 phase, increasing the population in this phase from 44 ± 5% (control) to 54 ± 6% (Figure 8g). Notably, treatment with 20 μg/mL of cD caused a significant drop in cell population as seen by flow cytometry. As a result, measurements at 50 and 100 μg/mL of cD could not be done, and only bD was tested at these doses. At the two highest doses, the effect of bD on the cell cycle was similar to that observed at 20 μg/mL. The S-phase population increased from 21 ± 7% (control) to 39 ± 5% at 50 μg/mL and to 40 ± 6% at 100 μg/mL of bD (Figure 8h,i).

### Western Blotting

In NIH/3T3 cells, cD activated the Nrf2 pathway in a dose-dependent manner. At 20 μg/mL Nrf2’s expression increased 9- fold compared to control, and at 50 and 100 μg/mL settled at 11- fold. bD also increased Nrf2’s expression, but only 4- fold at the highest dose (100 μg/mL). Both bD and cD also caused a mild, dose-dependent rise in HO-1 levels – 2-fold with bD and 4-fold with cD at 100 μg/mL. The expression of p65 was not affected by the two nanomaterials (Figures 9a and 10a).

**Figure 9:**
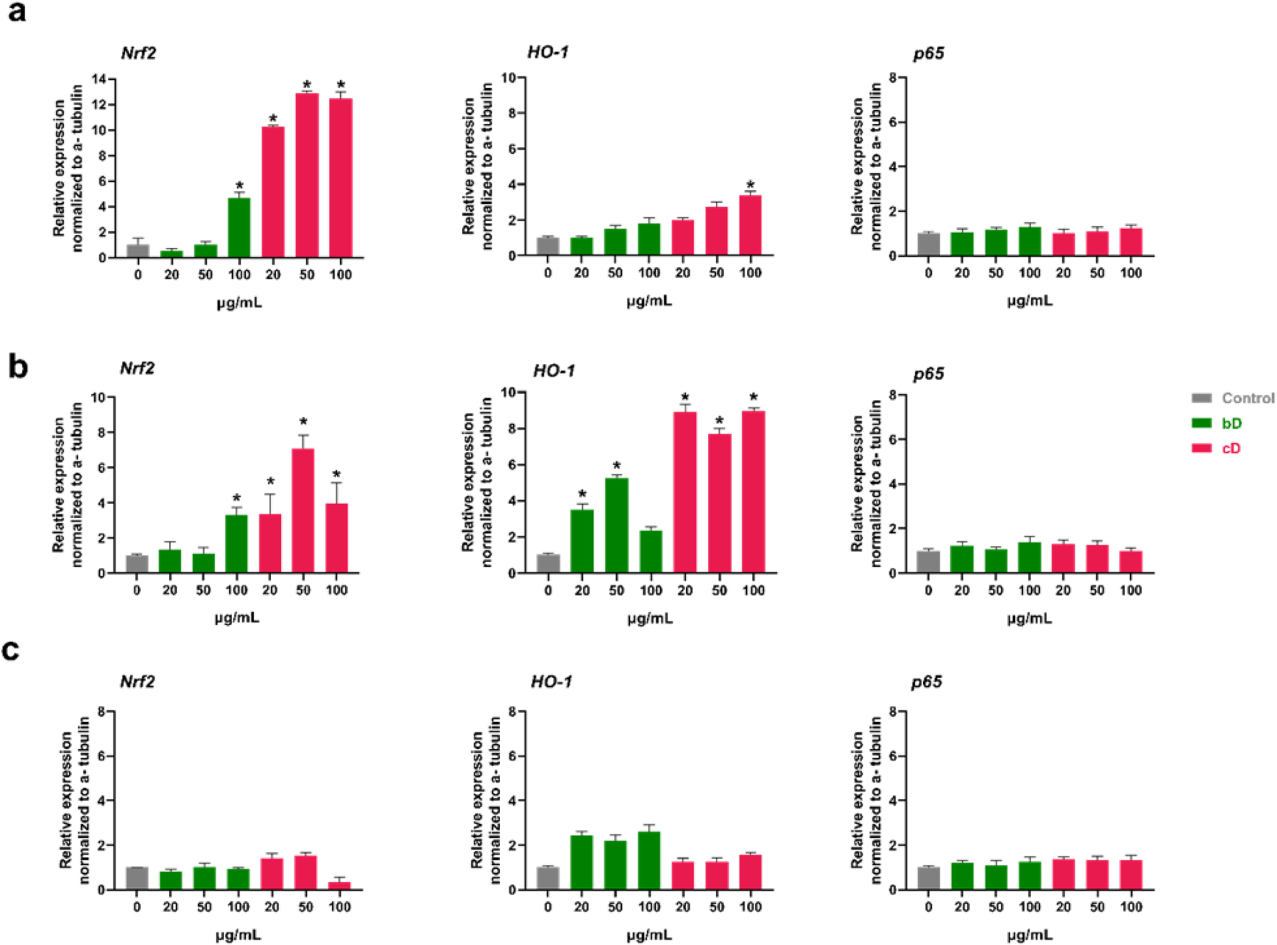
Relative expression of Nrf2, HO-1, and p65 in NIH/3T3 (a), HaCaT (b), and THP-1 (c) cells after treatment with 20, 50, and 100 μg/mL of bD or cD for 24 h.

**Figure 10:**
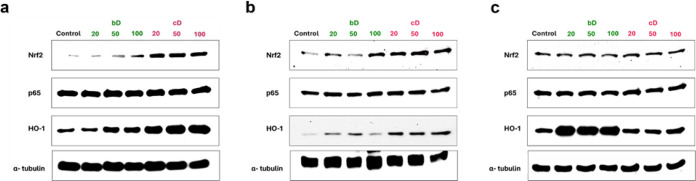
Western blots from NIH/3T3 (a), HaCaT (b) and THP-1 (c) cells after treatment with increasing doses of bD and cD for 24 h.

cD also activated the Nrf2 pathway in HaCaT cells. An increase in Nrf2 expression was observed at all tested doses, with the highest increase (by 6- fold) noted at 50 μg/mL. bD induced peak Nrf2 expression at 100 μg/mL, showing a 3- fold increase compared to control. Additionally, cD significantly increased HO-1 expression (by 8-fold) at all tested doses. bD also increased HO-1 expression, particularly at 20 and 50 μg/mL. The expression of p65 was not affected by the two nanomaterials (Figures 9b and 10b).

In THP-1 cells, bD had no effect on Nrf2 expression. At the highest dose (100 μg/mL), cD slightly reduced Nrf2 levels by 0.5- fold compared to control, but this change was not statistically- significant. cD did not affect HO-1 expression, while bD increased HO-1 levels by 1.5- fold at all tested doses. Similar to the findings in NIH/3T3 and HaCaT cells, neither bD nor cD affected p65 expression (Figures 9c and 10c).

## Discussion

Our results indicate that the synthesis of graphene is directly correlated with its biological effects. Specifically, we observed differences in the toxicity between biologically and chemically produced *N*- doped graphene with green graphene being more biocompatible at all cell lines than chemical graphene. Furthermore, our findings indicate that biological responses of graphene nanomaterials in living cells depend on the type of cells and tissues they interact with.

More specifically, in NIH/3T3 fibroblast cells, cD was toxic at all tested doses at the MTT assay. At low doses, bD was less toxic than cD but at higher doses (20–200 μg/mL) both nanomaterials were equally toxic to fibroblast cells, as viability reduced by 50%. Also, at the highest dose of 100 μg/mL, both nanomaterials affected the ability of cells to form colonies. However, the effect of cD was potent than that of bD, indicating that cD causes more serious long- term damage than its biologically- produced form. At this dose, both nanomaterials arrested the cell cycle in the S phase. The same effect as cD and bD on the cell cycle of fibroblast cells is also observed with GO. In a study by Hashemi et al., GO at a dose of 100 μg/mL arrested the cell cycle of embryonic fibroblasts in the S phase.^36^ During this phase, DNA synthesis occurs, which is a critical step for cell division and thus, interventions in this process usually have very poor outcomes. The family of GO nanomaterials has also been found in other studies to disrupt fibroblasts in the S phase through mechanisms that include ROS formation, DNA damage, and the induction of fragments in the DNA double helix.^37^ At this dose, cD also induced apoptosis in the cell population. This effect was not observed after treatment with bD.

The difference in toxicity between bD and cD became more evident in HaCaT keratinocytes. In the MTT assay, cD caused a substantially greater decrease in cell viability than bD, at both 24 and 48 h of incubation. Moreover, it should be noted that cD was toxic at all tested doses at both 24 and 48 h. Following 48 h of treatment with cD, cell viability declined significantly in a dose- dependent manner, reaching only 30% at 200 μg/mL. Pelin et al., also investigated the effects of chemically produced graphene- based nanomaterials in HaCaT keratinocytes. Similar to cD, treatment with increasing doses of few layers graphene (FLG) or GO for 48 h led to a significant dose- dependent decrease in cell viability, as measured by the WST-8 assay.^38^

Treatment with 50 and 100 μg/mL of cD for 48 h also had long- term effects on HaCaT keratinocytes, resulting in a significant reduction in colony formation, with a decrease of 50% at the highest tested dose. Pelin et al. also examined the effects of graphene nanomaterials on the proliferation of HaCaT cells. However, in contrast to our finding neither FLG nor GO influenced cell proliferation.^38^ Additionally, in our study, green *N*- doped graphene also did not significantly affect colony formation at any of the tested doses.

Although neither nanomaterial affected the cell cycle of HaCaT keratinocytes, cD induced high levels of apoptosis in the cell population. Moreover, cD significantly increased ROS formation in HaCaT keratinocytes, in a dose- dependent manner. At 20 μg/mL of cD, ROS production rose by 40% and at 100 μg/mL ROS production increased by 73%. Significant ROS production in HaCaT cells was also observed with FLG and GO in a separate study by Pelin et al. Treatment with 100 μg/mL of FLG led to a ROS production by 25%, while GO at the same dose altered ROS levels by 39%. The researchers noted that the elevated ROS levels contributed to mitochondrial dysfunction of HaCaT cells.^39^

It is important to note that bD did not induce ROS formation in any of the cell lines. On the contrary, other biologically produced graphene- based nanomaterials have been reported in literature to elicit an oxidative stress response. For example, biologically reduced rGO, synthesized with ecological methods, utilizing a polysaccharide from the wild mushroom *Pleurotus flabellatus Sacc* as the reducing agent (PR-GO), significantly affect ROS formation in human peripheral blood mononuclear cells (PBMCs). Although PR- GO did not reduce the viability of PBMCs, even at the maximum dose of 250 μg/mL, it caused a significant dose- dependent increase in ROS levels, which at the maximum dose, nearly doubled compared to control cells.^40^ An even greater induction of oxidative stress was observed in breast cancer cells, MCF-7 treated with bacterial- origin rGO. This rGO was synthesized using biomass from the bacterium *Bacillus marisflavi* as a reducing and stabilizing agent (B- rGO). Compared to chemically produced GO, B-rGO had a much stronger effect on the viability of MCF-7 and resulted in a more significant dose-dependent increase in ROS levels.^41^

The toxicity of bD and cD also exhibited significant differences in THP-1- derived macrophages. In MTT assay, treatment with cD for 48 h significantly reduced the viability of differentiated THP-1 macrophages in a dose-dependent manner. At the highest dose of 200 μg/mL, cell viability decreased to 50%. Other chemically produced graphene- based nanomaterials also exhibited similar toxicity to macrophages cells. For example, Duan et al. performed *in vitro* evaluation of the toxicity of GO nanosheets on Raw264.7 macrophage cells. The GO nanosheets significantly reduced cell viability in a time- and dose- dependent manner. After 24 h, cell viability dropped to 45% at 10 μg/mL and to 35% at 200 μg/mL.^42^ In contrast, bD did not induce significant toxicity in macrophages cells at any of the tested doses.

cD also significantly increased apoptosis and necrosis in the cell population in a dose- dependent manner, a result that was not observed with bD. Similar results were obtained by the research team of Yan et al., who also studied the differences in the biological responses of biologically- and chemically- produced graphene nanomaterials. The researchers synthesized graphene quantum dots (GQDs) using only glucose in an aqueous solution (HGQDs). Compared to GQDs synthesized through the conventional chemical method (CGQDs), the HGQDs exhibited significant greater biocompatibility with THP-1 macrophage cells. In apoptosis/necrosis assessments, HGQDs did not cause any changes in the population of THP-1 cells after either 6 or 12 h of incubation. In contrast, CGQDs significantly and time-dependently increased apoptosis in the cell population, with a 59.4% rise in apoptosis observed after 12 h. The two types of GQDs also had different effects on the viability of human umbilical vein endothelial cells (HUVECs). At all tested doses, which were quite high (100–500 μg/mL), HGQDs did not induce toxicity in HUVECs, with cell viability remaining above 80%. On the contrary, at the same doses, CGQDs significantly reduced cell viability to levels below 40%.^43^ bD and cD had also different influences on the cell cycle of THP-1 derived macrophages. The biologically produced bD increased the population of cells in the S phase, while the chemical cD significantly reduced the cells in this phase, arresting the cell cycle at the G0/G1 phase. It appears that cD caused DNA damage that prevented entry into the S phase, thereby inhibiting DNA synthesis.

The diversity in the biological responses of the two nanomaterials across the three different cell lines was also confirmed through the analysis of the relative expression of proteins involved in two known intracellular signaling pathways. The primary goal of this study was to evaluate the toxicity of the two graphene compounds; therefore, we chose to study the three main representatives of the signaling pathways of Nrf2 and p65 (NF-kB). Both pathways are activated in oxidative stress and inflammation. Nrf2 is the master regulator of the antioxidant system, and it is influencing among others, cell fate, cell proliferation, cell differentiation and apoptosis. It can also determine resistance to treatment, aging processes and the progression of infections. Nrf2’s deregulation appears to affect the immune system response.^44^ For these reasons, the activation of keap1/Nrf2/ARE pathway is recognized as a robust cytoprotective mechanism in cells, and its induction may play a significant role in the treatment of various pathologies. The main regulator of Nrf2 activity is apparently the protein keap1.^45^ However, Nrf2 is quite unstable and thus it is also being regulated by a few different proteins, many of which participate in other major signaling pathways (e.g., the PI3K/AKT).^44,46^ One of these signaling pathways that affect the fate of Nrf2 is that of p65. Although the interaction between the Nrf2 and p65 pathways has not been fully elucidated yet, it appeared that both pathways strongly interact under oxidative stress conditions.

Nanomaterials can initiate inflammation by inducing oxidative stress or may exhibit antioxidant properties. The literature contains only a few reports on the effects of graphene nanomaterials on the signaling pathways related to inflammation and oxidative stress. In our study, bD and cD affected both pathways differently, and the responses of the two compounds varied across different cell types.

In NIH/3T3 cells, all doses of cD and the highest dose of bD significantly altered Nrf2’s expression and had an impact in its cathodic target, HO-1. It is important to note that this effect was also dose- dependent on both nanomaterials. However, the effect of cD was significantly stronger than that of bD. These results align with those of our previous study. In NIH/3T3 cells, plain graphene exfoliated using toxic solvent DMF, was found to have a stronger effect in Nrf2 pathway than biologically- exfoliated biographene.^12^ In the cytotoxic analysis, cD was found to be toxic in fibroblasts at all doses, reducing significantly cell viability and increasing apoptosis of NIH/3T3 cells in a dose- dependent manner. High doses of cD also caused changes in the cell cycle, arresting cells in S phase. Considering these results, we believe that its strong toxicity likely initiated inflammatory processes in fibroblasts, promoting the activation of Nrf2 as a protective mechanism.

In HaCaT cells, cD had a similar effect on Nrf2’s signaling as it did in fibroblasts, causing a significant dose- dependent increase in the expression of Nrf2 and HO-1. In these cells, cD also induced significant ROS formation and apoptosis in the cell population. It appears that excess ROS production led to oxidative stress in HaCaT cells, which activated the Nrf2 pathway. As with fibroblasts, mild activation of the pathway was also observed in HaCaT cells with the biological form, bD, but at much lower levels than cD.

In THP-1 cells, an increase in HO-1 relative expression was observed only after treatment with the biological form, bD. This increase did not seem to correlate with an increase in total Nrf2 expression. Given that the nanomaterials were administered to cells for 24 h, it is possible that during this time, bD activated Nrf2, allowing it to enter the nucleus and contributed to the increase of HO-1. Further analysis of the nuclear and cytosolic expression of the Nrf2 and HO-1 proteins is needed to better evaluate this effect. But, since “green” *N*- doped graphene did not induce oxidative stress nor cause significant cell mortality in cytotoxicity assays, the increased expression of HO-1 could indicate the antioxidant properties of this nanomaterial, potentially through different signaling pathways. Another noteworthy observation is that in macrophages cells, treatment with cD resulted in the highest rates of cell death and induced significant changes in the cell cycle, preventing progression to the S phase. As cD did not activate either Nrf2 or p65, it likely acted on these cells through alternative mechanisms, such as the activation of apoptotic signaling pathways.

## Conclusions

In our study we evaluated the biological responses of *N*- doped graphene, *in vitro*, in three different cell lines. The objective of this study was to assess whether “green” chemistry can increase the biocompatibility of *N*- doped graphene and thus alter its clinical value in biomedical applications.

The exfoliation strategy not only alters the morphology and interlayer architecture of N-doped graphene but also affects surface charge a critical factor for dispersion stability and biocompatibility. The smaller fragment size, reduced *d*-spacing, and heterogeneous surface profile of bD may underlie its more favorable biological performance, as observed throughout this study.

The cD had slightly more negative charge than bD (−7.5 mV versus −1.5 mV) but both nanoparticles fall in the range ± 10 mV and can be characterized as neutral. Thus, although different the effect of charge in cells might not be significant. The size and shape and surface characteristics are other factors that can affect uptake and toxicity. Doping graphene with nitrogen has been reported to reduce cytotoxicity compared to pristine graphene^47^ and this effect is attributed to modifications in the shape and edges of the graphene sheets^48^ and the smaller size^49^ of doped graphene which may reduce physical damage and oxidative stress. This is supported by our data, which shows ROS formation from cD but not from bD. Lower oxidative stress also contributes to a diminished inflammatory response. Additionally, nitrogen doping enhances the hydrophilicity of graphene, thereby improving its dispersibility in aqueous biological environments.^50^ Moreover, green production of graphene, such as the one proposed in the current article, could provoke less toxicity to cells than chemically synthesized graphene potentially due to the presence of impurities (solvents, or acidic byproducts) in the chemically synthesized graphene.

The type of cell can significantly influence the effect of nanoparticle charge. In the present study, three cell types were used: NIH/3T3 fibroblasts, HaCaT epithelial cells, and THP-1 macrophages. The membrane surface charge of these cells may affect their interaction with the nanoparticles bD and cD. Previous studies suggest that HaCaT cells possess a more strongly negative surface charge, making them less susceptible to negatively charged nanoparticles.^51^ However, direct measurements of membrane zeta potential are not consistently reported across studies, making it difficult to quantitatively compare their interactions with bD and cD. Despite this limitation, our findings indicate that cellular responses to neutral nanoparticles such as bD and cD can vary. Specifically, HaCaT cells may permit easier penetration of these neutral particles due to their unique membrane characteristics.

Chemically produced *N*- doped graphene was more toxic than green *N*- doped graphene across all *in vitro* assays. In NIH/3T3 cells, cD activated the cytoprotective Nrf2 signaling pathway whereas in HaCaT cells it triggered oxidative responses, significantly altering ROS production and increasing the apoptosis of the population. In THP-1 derived macrophages cD acted through different mechanisms, increasing apoptosis and necrosis of cells and interrupting the cell cycle in G0/G1 phase. Although high doses of bD were also toxic to all three cell lines, its effects were overall significantly milder compared to those of cD.

The interactions of both *N*- doped graphene compounds with other critical intracellular signaling pathways involved in other processes (i.e., apoptosis) will be investigated in the future as this will provide new insights into the biological actions of these nanomaterials. Additionally, it is important to investigate the localization of nanomaterials within the cells, as this often appears to be related to their toxicity. Finally, *in vivo* evaluation of the toxicity of both chemical and biological nanomaterials is necessary before their use in medical applications.

In conclusion, we demonstrated that the synthesis of graphene-based nanomaterials using “green” exfoliation techniques preserves their uniqueness and functional characteristics while simultaneously reducing their negative effects on cells. Therefore, “green” *N*-doped graphene emerges as an ideal candidate for biomedical applications.

## Abbreviations

bD: biologically exfoliated *N*- doped graphene
BSA: bovine serum albumin
cD: chemically exfoliated *N*- doped graphene
DMEM: Dulbecco’s Modified Eagle’s Medium
DMSO: dimethyl sulfoxide
EG: expanded graphite
FBS: fetal bovine serum
FLG: few- layers graphene
GO: graphene oxide
GQDTs: graphene quantum dots
HO-1: heme oxygenase 1
HUVECs: human umbilical vein endothelial cells
MFI: mean fluorescence intensity
N: nitrogen
NF- κΒ: nuclear factor- κB
Nrf2: nuclear factor (erythroid-derived 2)- like 2
PBMCs: peripheral blood mononuclear cells
PI: propidium iodide
rGO: reduced graphene oxide
ROS: reactive oxygen species
Rpm: revolutions per minute
Rps: revolutions per second
RT: room temperature
SF: surviving fraction
